# Differences in Smoking Prevalence Between the Adult Tobacco Survey and the Behavioral Risk Factor Surveillance System

**Published:** 2004-09-15

**Authors:** Leigh T Ramsey, Andrew Pelletier, Susan Knight

**Affiliations:** New Hampshire Department of Health and Human Services, Centers for Disease Control and Prevention, Atlanta, Ga; New Hampshire Department of Health and Human Services, Centers for Disease Control and Prevention, Atlanta, Ga; New Hampshire Department of Health and Human Services Concord, NH

To the Editor:

Smoking prevalence is a principal outcome for evaluating tobacco-control efforts, but prevalence estimates in New Hampshire differed between two surveys conducted during 2002. Smoking prevalence was 17.9% (95% confidence interval [CI] 16.3%–19.5%) in the Adult Tobacco Survey (ATS) and 23.2% (95% CI 21.8%–24.5%) in the Behavioral Risk Factor Surveillance System (BRFSS) (absolute percentage point difference = 5.3%; relative percentage difference = 22.8% [5.3%/23.2%]). We examined possible reasons for this observed difference.

The ATS and BRFSS were both developed by the Centers for Disease Control and Prevention. The ATS included 103 questions related to knowledge, attitudes, and behaviors regarding tobacco; the BRFSS included 138 questions related to health behavior risk factors, including 11 questions about tobacco, which followed questions on nine other health topics. The same survey research firm administered both surveys, which were population-based, random-digit-dialed telephone surveys of noninstitutionalized adults aged 18 or older. Smoking prevalence for both surveys was determined by the number of persons who had smoked at least 100 cigarettes in their lifetime and were current smokers. The introduction to the ATS informed potential participants that it was a survey on health and tobacco; the BRFSS was introduced as a survey on health and health practices.

The ATS was conducted in August and September with a Council of American Survey Research Organizations (CASRO) response rate of 52.6% and a sample size of 3000; the BRFSS was conducted throughout the year with a CASRO response rate of 53.2% and a sample size of 5039. No monthly variation existed in smoking prevalence within surveys. The demographics of respondents were similar in both surveys (Table) (1,2).

Given the similarities between the surveys, possible causes of the discrepancy in estimated smoking prevalence between the ATS and the BRFSS in New Hampshire are differences in the survey introduction and differences in question placement. One study conducted in California suggested the tobacco-specific introductory statement in the ATS may have caused certain smokers to deny tobacco use (3). State health departments that conduct the ATS and the BRFSS should be aware of potential differences in smoking prevalence between these two surveys and be prepared to address these differences when communicating with the public and policy makers. Further research is needed to determine if differences in smoking prevalence between these two surveys exist in other states.

## Figures and Tables

**Table:** Demographics of Respondents to Adult Tobacco Survey and Behavioral Risk Factor Surveillance System (Data Unweighted), New Hampshire, 2002

	**Adult Tobacco Survey** **(n = 3000)**	**Behavioral Risk Factor Surveillance System** **(n = 5039)**

	**%**	**%**

**Age (years)**		

18-24	5.8	6.1
25-34	16.2	15.2
35-44	24.0	24.3
45-54	21.1	21.5
55-64	15.0	15.0
⩾65	17.9	18.0

**Years of education**		

<12	5.5	6.7
12	28.1	29.5
13-15	26.1	26.4
⩾16	40.3	37.3

**Income ($)**		

<10,000-14,999	6.8	7.7
15,000-24,999	12.4	13.8
25,000-49,999	31.1	32.7
⩾50,000	49.6	45.9

**Sex**		

Male	42.0	42.0
Female	58.0	58.0

**Race**		

White	93.9	95.4
